# Unveiling the molecular toxicity of plasticizers derived from microplastics (DMP and DEP) in diabetic kidney disease: integrative insights from network toxicology and multi-omics analysis

**DOI:** 10.3389/fimmu.2026.1918728

**Published:** 2026-08-26

**Authors:** Kaifeng Xie, Jiasheng Huang, Shiyun Ling, Xinyu Shi, Hesheng Li, Renfa Huang, Hanli Lin

**Affiliations:** 1 Nephropathy Department, Shenzhen Hospital (Futian) of Guangzhou University of Chinese Medicine, Shenzhen, Guangdong, China; 2 Research Institute, Shenzhen Hospital (Futian) of Guangzhou University of Chinese Medicine, Shenzhen, Guangdong, China

**Keywords:** diabetic kidney disease, molecular docking, molecular dynamics simulation, network toxicology, phthalate plasticizers

## Abstract

**Background:**

Diabetic kidney disease (DKD) is a prevalent microvascular complication with limited therapeutic options. This study aimed to identify biomarkers associated with microplastic-associated plasticizers in DKD and to explore their potential molecular mechanisms.

**Methods:**

Publicly available datasets were integrated for biomarker discovery, followed by experimental validation in human proximal tubular epithelial cells (HK-2). Pathway enrichment and immune infiltration analyses were performed to characterize the biological relevance of the identified biomarkers. Molecular docking and molecular dynamics simulations were used to evaluate the predicted interactions between the biomarkers and the plasticizers dimethyl phthalate (DMP) and diethyl phthalate (DEP). Single-cell transcriptomic analysis was further conducted to characterize the cell-type-specific expression of the identified biomarkers.

**Results:**

CASP3, PTGES, and SLC6A2 were identified as candidate biomarkers. Pathway analysis revealed that these genes were notably enriched in oxidative phosphorylation, while immune infiltration analysis indicated a strong correlation between PTGES and memory B cells. Molecular docking and molecular dynamics simulations predicted stable interactions between the biomarkers and DMP and DEP. *In vitro* experiments further showed that DMP and DEP exposure reduced HK-2 cell viability, promoted apoptosis, and dysregulated the expression of CASP3, PTGES, and SLC6A2. Notably, these toxic effects were exacerbated under high-glucose conditions, suggesting an enhanced combined effect between the diabetic milieu and plasticizer-induced stress. Single-cell transcriptomic analysis further indicated predominant CASP3 expression in proximal convoluted tubule (PCT) cells.

**Conclusions:**

This study provides a hypothesis-generating framework by identifying CASP3, PTGES, and SLC6A2 as potential DKD biomarkers that are computationally predicted and experimentally shown to be regulated by microplastic-associated plasticizers. These findings suggest potential molecular links between plasticizer exposure and DKD-related cellular injury; however, their exposure-dependent relevance and mechanistic roles in human DKD require further validation.

## 1 Introduction

Diabetic kidney disease (DKD) is a common microvascular complication of diabetes, affecting approximately 40% of individuals with diabetes, and represents a leading cause of disability and mortality in this population (1). DKD has emerged as the primary driver of end-stage renal disease (ESRD) worldwide, exerting a particularly heavy burden on low-income nations and socioeconomically disadvantaged communities (2). The global diabetic population currently stands at 564 million and is projected to rise to 600 million by 2035 and 783 million by 2045 (3, 4). The pathogenesis of DKD involves a complex interplay of hemodynamic alterations, metabolic dysregulation, inflammation, and fibrosis (5). Nevertheless, despite substantial renoprotective advances achieved with sodium-glucose cotransporter 2 (SGLT2) inhibitors, a considerable residual risk of DKD progression persists—particularly in certain patient subgroups—highlighting the need to explore additional modifiable risk factors such as environmental toxicants (6). Therefore, gaining more comprehensive insight into the fundamental mechanisms of this disease is crucial for devising innovative interventions to slow progression and minimize related complications.

Microplastics (MPs), defined as plastic particles smaller than 5 mm, have emerged as a growing environmental concern due to potential health risks (7). MPs may act as vectors for environmental pollutants, and specifically, interactions between MPs and associated additives depend on physicochemical properties (8). Low-log K_ow_ phthalates such as DMP(≈1.15) and DEP(≈2.38) readily leach from polymer matrices and mainly exist as free chemicals, whereas more hydrophobic phthalates such as DBP(≈4.45) and DEHP(≈7.50) remain associated with MP surfaces and co-migrate with particles (9). Among the detectable additives, acetyl tributyl citrate (ATBC) and several phthalate esters-including dimethyl phthalate (DMP), diethyl phthalate (DEP), and dioctyl phthalate (DOP)-have attracted attention (10). Experimental studies suggest that these plasticizers may contribute to the progression of DKD, including disruption of gene expression, induction of oxidative stress, activation of inflammatory responses, and mitochondrial dysfunction (11, 12). Given the close association between DKD pathogenesis and metabolic disturbances, including insulin resistance, lipid metabolism dysregulation, and chronic low-grade inflammation (13), the metabolic effects of plasticizers are concerning. Phthalates have been shown to promote adipocyte differentiation and visceral fat accumulation via activation of the PPARγ signaling pathway (14), and to disrupt thyroid hormone signaling and mitochondrial energy metabolism, thereby exacerbating insulin resistance (15) and DKD progression. For instance, exposure to triphenyl phosphate (TPHP) induces endoplasmic reticulum stress, leading to hepatic insulin resistance and diabetic phenotypes, as well as renal structural damage in animal models (16). Collectively, microplastic-associated plasticizers may promote DKD progression through direct nephrotoxicity, systemic inflammatory activation, endocrine disruption, and metabolic dysregulation. Although large-scale prospective epidemiological studies remain limited, experimental evidence suggests that these environmental pollutants warrant consideration in chronic kidney disease prevention strategies, under widespread environmental exposure. Recent reviews have systematically summarized the nephrotoxic mechanisms of microplastics, including the induction of oxidative stress, inflammatory responses, mitochondrial dysfunction, and ferroptosis in renal cells, as well as the potential synergistic toxicity of co-exposure to microplastics and additives such as plasticizers, all of which may contribute to the development and progression of chronic kidney disease (17). It is important to note that microplastics may induce toxicity through both particle-mediated effects and associated chemical additives. This study focused specifically on the chemical toxicity of the plasticizers DMP and DEP in dissolved form—compounds that originate from plastic additives and can leach from microplastic particles.

Network toxicology is an emerging systems-level approach that integrates network pharmacology and informatics, leveraging high-throughput technologies such as proteomics and genomics to map compound-target-pathway interactions and predict potential toxic mechanisms (18). Molecular docking simulates the spatial configuration and binding interactions between small molecules and target proteins at the atomic level (19). In toxicological research, molecular docking helps predict and elucidate interactions between toxins and biomolecules, providing mechanistic insights into potential toxicity (20).

Based on publicly available DKD datasets, this study integrated single-nucleus RNA sequencing, bulk transcriptomic analysis, and network toxicology approaches to identify biomarkers potentially associated with microplastic-associated plasticizers in DKD. The identified candidates were further characterized through analyses of biological functions, immune microenvironment interactions, regulatory networks and molecular dynamics behavior. A stepwise integrative strategy combining computational prediction with experimental evaluation in a disease-relevant cellular model was employed to provide complementary support for associative bioinformatic findings and preliminarily assess the effects of microplastic-associated plasticizers on DKD-related renal tubular injury. Collectively, this comprehensive analysis aimed to explore the potential molecular links between microplastic-associated plasticizers and DKD progression, while identifying candidate binding targets for future investigation. As an initial step, this study provides computational and *in vitro* evidence, with *in vivo* validation still needed.

## 2 Materials and methods

### 2.1 Data sources and target acquisition

Transcriptomic datasets were obtained from the Gene Expression Omnibus (GEO) database (https://www.ncbi.nlm.nih.gov/geo/). GSE96804 (GPL17586; 41 DKD vs 20 controls, glomerular tissue) served as the discovery set; GSE30122 (GPL571; 19 DKD vs 50 controls) served as validation set, respectively; GSE131882 (GPL24676; 3 DKD vs 3 controls, single-nucleus RNA-seq) was used for cell-type characterization. The GSE131882 dataset comprises only 3 donors per group; all downstream single-cell findings should therefore be regarded as exploratory and hypothesis-generating pending replication in larger cohorts.

Microplastic-associated plasticizer targets were retrieved by querying SMILES notations of dimethyl phthalate (DMP), diethyl phthalate (DEP), acetyl tributyl citrate (ATBC), and dioctyl phthalate (DOP) — sourced from PubChem — against the SwissTargetPrediction database (human proteins only) using default parameters (probability > 0). Gene names were standardized using UniProt. DKD-associated target genes were retrieved from GeneCards using the search term “Diabetic Kidney Disease”.

### 2.2 Biomarker identification and predictive modeling

Differentially expressed genes (DEGs) between DKD and control samples in GSE96804 were identified using the limma package (v3.19) with thresholds of P < 0.05 and |log2FC| > 0.5. Volcano plots and heatmaps of top DEGs were generated using ggplot2 (v3.4.4) and ComplexHeatmap (v2.18.0), respectively.

Key genes were obtained by intersecting microplastic-associated plasticizer-related targets (Section 2.1) with DKD-related genes using ggVennDiagram (v0.1.10), and further intersected with DEGs. Functional enrichment (GO and KEGG, P < 0.05) was performed using clusterProfiler (v4.10.0), with top GO terms (by gene count) and top 10 KEGG pathways visualized. PPI networks were constructed using STRING (confidence > 0.4) and visualized in Cytoscape (v3.10.2). Toxicity prediction of ATBC, DMP, DEP, and DOP was performed using ProTox 3.0.

Candidate biomarkers were identified by intersecting LASSO-selected genes (glmnet v4.1-8; non-zero coefficients) and Boruta-confirmed genes (Boruta v8.0.0; seed = 7221108, P < 0.01, maxRuns = 300). Final biomarkers were defined based on consistent differential expression (Wilcoxon P < 0.05) in both GSE96804 and GSE30122 datasets. Diagnostic performance was evaluated using ROC analysis (pROC v1.18.0; AUC > 0.7).

A nomogram model was constructed using rms (v6.7-1). Model performance was assessed via ROC (AUC > 0.7, 95% confidence interval (CI)), calibration curve (Hosmer–Lemeshow test, P > 0.05), and decision curve analysis (rmda v1.6). Internal validation included bootstrap resampling (1,000 iterations) to estimate optimism-corrected AUC and repeated 5-fold cross-validation (50 repeats) using caret. External validation was performed using independent dataset GSE30122.

### 2.3 Functional and immune characterization

Biomarker-associated pathways were explored using pre-ranked GSEA based on Spearman correlations between CASP3, PTGES, SLC6A2 and all genes in the GSE96804 dataset (cor and cor.test in R), with no correlation threshold applied. Genes were ranked in descending order to perform GSEA using clusterProfiler (v4.10.0) gseKEGG with MSigDB KEGG gene set “c2.cp.kegg.v2023.1.Hs.symbols.gmt” as reference. Significant pathways were defined as |NES| > 1 and P < 0.05, and the top 5 pathways ranked by P-value were visualized.

Immune infiltration was assessed using ssGSEA in GSVA (v1.46.0) with immune cell marker sets from Charoentong et al. (21), generating relative enrichment scores for 28 immune cell types. Group differences between DKD and controls were evaluated using Wilcoxon tests with BH correction (P_adj < 0.05). Spearman correlation analyses were further performed between differentially infiltrated immune cells, biomarker expression, and immune-related genes, including chemokines, receptors, and MHC genes retrieved from TISIDB, with thresholds |cor| > 0.30 and P < 0.05.

### 2.4 Molecular docking and dynamics simulation

Phthalate structures were obtained from PubChem and target protein structures from the PDB database. Molecular docking was performed using CB-Dock2, and complexes with binding affinity ≤ −5 kcal/mol were selected for further analysis.

To validate docking stability, molecular dynamics (MD) simulations were conducted using GROMACS 2024.2. Protein and ligand parameters were described using the AMBER14SB and GAFF force fields, respectively, with ligand charges assigned via the Antechamber/BCC method. Systems were solvated in a TIP3P cubic water box under periodic boundary conditions, and neutralized with 0.15 mol/L Na^+^/Cl^-^ ions. Energy minimization was performed using the steepest descent algorithm (Fmax < 1000 kJ/mol/nm), followed by NVT (300 K, 100 ps, V-rescale) and NPT (1 bar, 100 ps, Parrinello–Rahman) equilibration. Production MD simulations were run for 100 ns with a 2 fs timestep using the LINCS constraint algorithm. Structural stability was evaluated using RMSD, RMSF, and radius of gyration (Rg).

### 2.5 Single-cell RNA sequencing analysis

The GSE131882 dataset was processed using the Seurat package (v5.0.1). Quality control was applied with thresholds of 500 < nFeature_RNA < 4,000, nCount_RNA < 16,000, and percent.mt < 80% (22). After sample merging and normalization, highly variable genes (top 2,000) were identified for downstream analysis. Principal component analysis (top 30 PCs) was performed for dimensionality reduction, followed by t-SNE clustering (resolution = 0.6). Cell types were annotated based on marker genes reported in the original study (23).

Cell–cell communication analysis was performed using CellChat (v1.6.1), and aggregated interaction networks were computed using the aggregateNet function to quantify interaction numbers and strengths between cell types. Key cells were defined based on high biomarker expression across cell populations.

To investigate cellular heterogeneity and developmental trajectories, key cells were re-clustered and visualized using UMAP. Pseudotime trajectory analysis was performed using Monocle (v2.26.0) to infer differentiation states, with the trajectory root automatically determined by Monocle’s default algorithm based on cell density distribution in the reduced dimensional space, without manual specification. Biomarker expression dynamics across trajectories were visualized using heatmaps. To validate the biological direction of the trajectory, the expression trends of proximal tubule maturation markers (SLC34A1, LRP2) and injury markers (HAVCR1, LCN2) were examined along pseudotime.

### 2.6 *In vitro* experimental procedures

The Human proximal tubular epithelial cell (HK-2 cell) line was selected for experimental validation because proximal tubular epithelial cells are key effectors in DKD progression and represent a primary target of environmental toxicants in the kidney. HK-2 cells were obtained from the American Type Culture Collection (ATCC, Manassas, VA, USA). HK-2 cells were cultured in DMEM/F12 supplemented with 10% FBS and 1% penicillin-streptomycin at 37 °C under 5% CO_2_. HK-2 cells were cultured under either normal-glucose conditions (NG; 5.5 mM D-glucose) or high-glucose conditions (HG; 30 mM D-glucose). Cells were preconditioned under the indicated glucose conditions for 24 h before treatment with DMP and/or DEP.

DMP (MCE, HY-N7106) and DEP (Sigma, 524972-5ML) were dissolved in DMSO (final concentration ≤0.1%, v/v). Based on preliminary dose–response experiments, concentrations of 10 and 30 μM were selected as the low and high doses, respectively. Under both NG and HG conditions, cells were assigned to six treatment groups: vehicle control, DMP-L (10 μM DMP), DMP-H (30 μM DMP), DEP-L (10 μM DEP), DEP-H (30 μM DEP), and DMP-H + DEP-H (30 μM DMP plus 30 μM DEP). Cells were exposed to the indicated treatments for 24 h unless otherwise specified.

Cell viability was assessed using CCK-8 (Mei5bio, MF128-Pro-01) with absorbance measured at 450 nm, and apoptosis was analyzed using Annexin V-FITC/PI staining (Solarbio, CA1020) followed by flow cytometry (BD FACSCalibur) and FlowJo analysis.

Total RNA was extracted using TRIzol and reverse transcribed using M5 First Strand cDNA kit (Mei5bio). Gene expression was quantified by qRT-PCR using 2^-^ΔΔCt with GAPDH as reference. Protein expression was evaluated by Western blot after RIPA extraction and SDS-PAGE transfer to PVDF membranes. Primary antibodies against CASP3 (1:2000, ab184787, Abcam; total CASP3, 32 kDa), PTGES (1:2000, 67736-1-Ig, Proteintech), SLC6A2 (1:1000, MA5-24647, Thermo Fisher), and GAPDH (1:1000, ab8245, Abcam) were used, and signals were detected by ECL and quantified using ImageJ. Primer sequences are listed in **Supplementary Table 1**.

### 2.7 Statistical analysis

Bioinformatics analyses were performed in R v4.2.2; group differences were assessed using the Wilcoxon test. For experimental data, all assays were performed in at least three independent replicates; results are expressed as mean ± SD. Multi-group comparisons were analyzed by one-way ANOVA followed by Tukey’s *post hoc* test (GraphPad Prism 8.0). P < 0.05 was considered statistically significant.

## 3 Results

### 3.1 Identification of CASP3, PTGES, and SLC6A2 as plasticizer-associated DKD biomarkers

**Figure 1:**
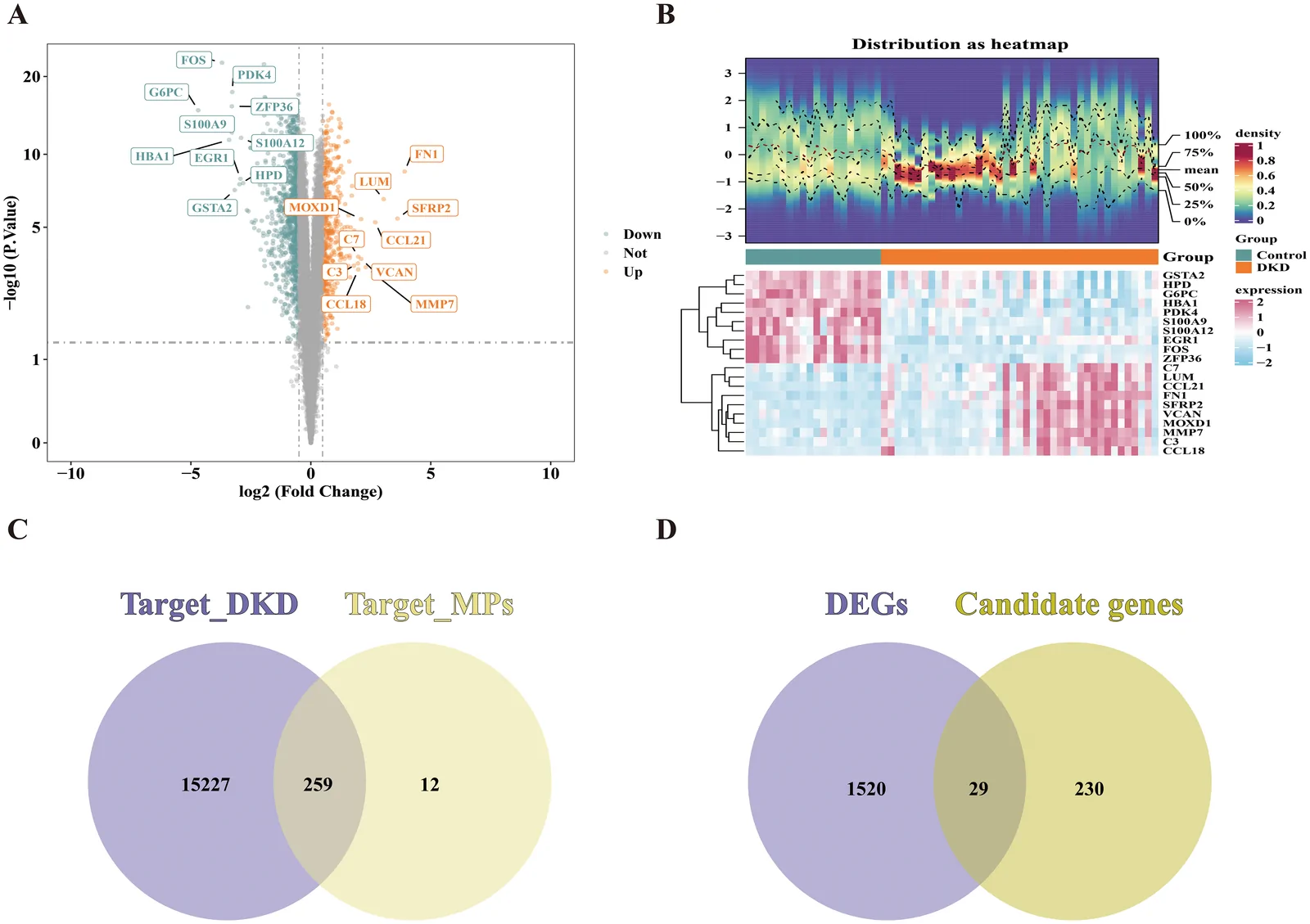
Identification of key genes associated with DKD and microplastic-associated plasticizers. **(A)** Volcano plot showing differentially expressed genes (DEGs) between DKD and control samples. **(B)** Heatmap of the top 10 upregulated and top 10 downregulated DEGs. **(C)** Venn diagram showing the overlap between microplastic-associated plasticizer-related targets and DKD-related genes. **(D)** Venn diagram showing the intersection of candidate genes and DEGs, resulting in 29 key genes. DKD, diabetic kidney disease; DEGs, differentially expressed genes.

In the GSE96804 dataset, 1,549 DEGs were identified, including 466 upregulated and 1,083 downregulated genes in DKD (**Figures 1A, B**). SwissTargetPrediction returned 100 predicted targets per compound (DMP, DEP, ATBC, and DOP), which were combined and deduplicated to yield 271 microplastic-associated plasticizer-related targets (**Supplementary Table 2**). Intersecting these with 15,486 DKD-related genes (**Supplementary Table 3**) yielded 259 candidate genes (**Figure 1C**), which were further refined to 29 key genes by intersecting with DEGs (**Figure 1D**).

Functional enrichment analysis of these key genes revealed 745 GO terms and 103 KEGG pathways, mainly involving processes such as cellular component disassembly and endopeptidase activity, as well as pathways including apoptosis and osteoclast differentiation (**Supplementary Figure 1A, B**; **Supplementary Tables 4**, **5**). PPI analysis identified interactions among 22 proteins, including PTGES and HPGDS (**Supplementary Figure 1C**). Toxicity prediction of ATBC, DMP, DEP, and DOP suggested generally lower nephrotoxicity compared with other toxicity categories (**Supplementary Figure 1D**; **Supplementary Table 6**), supporting their potential indirect renal effects.

**Figure 2:**
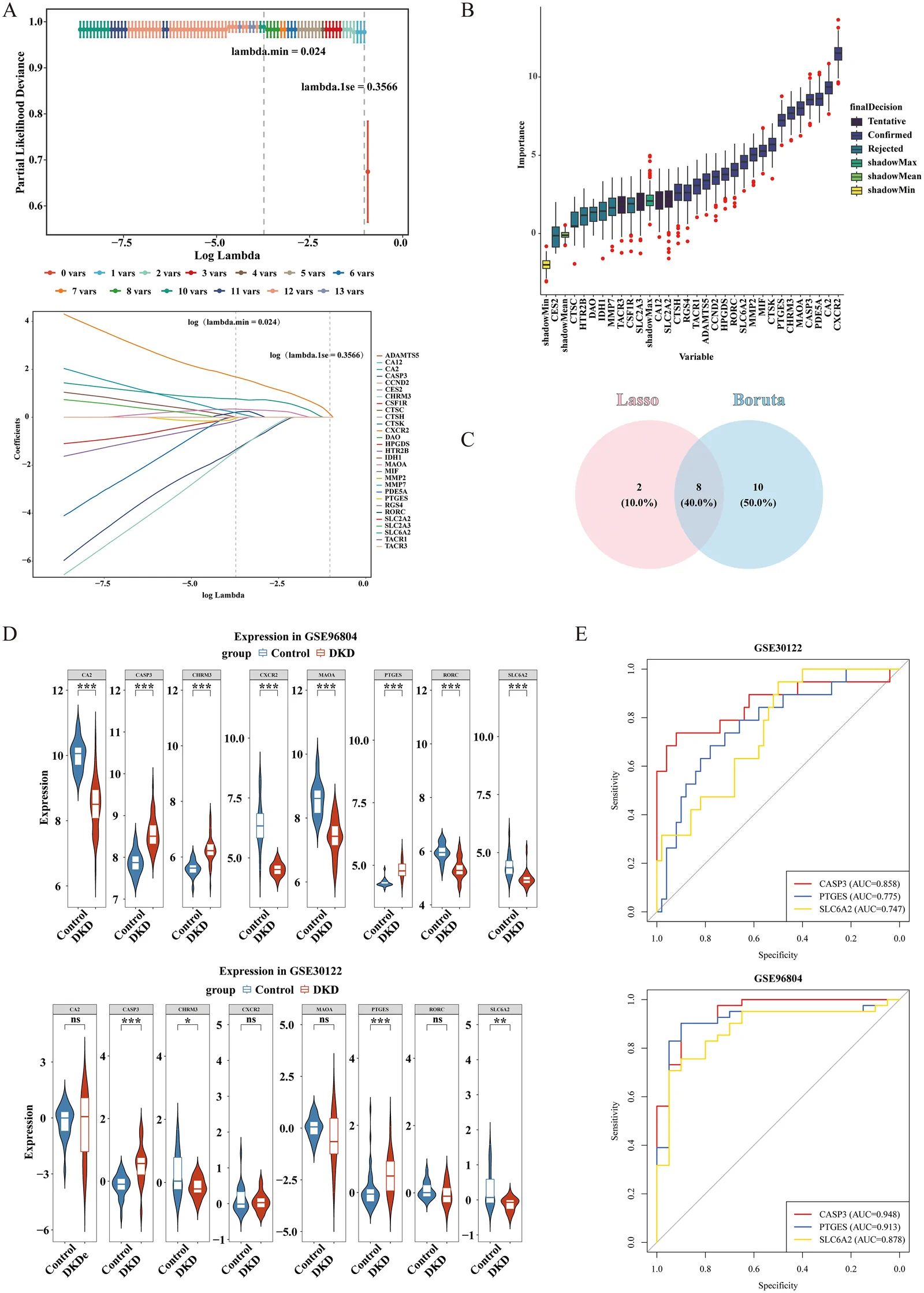
Identification of candidate biomarkers. **(A)** LASSO regression analysis for feature gene selection. **(B)** Boruta algorithm results for feature gene identification. **(C)** Venn diagram of overlapping genes identified by LASSO and Boruta analyses. **(D)** Expression levels of CASP3, PTGES, and SLC6A2 in DKD and control samples. **(E)** Receiver operating characteristic (ROC) curves evaluating the diagnostic performance of the biomarkers. LASSO, least absolute shrinkage and selection operator; ROC, receiver operating characteristic; AUC, area under the curve; DKD, diabetic kidney disease. *P < 0.05, **P < 0.01, ***P < 0.001, and ns, not significant.

Feature selection identified 10 genes via LASSO regression (λmin = 0.024) and 18 genes via Boruta analysis, yielding 8 intersecting candidates: CA2, CASP3, MAOA, CHRM3, RORC, CXCR2, PTGES, and SLC6A2 (**Figures 2A–C**). Expression validation in GSE96804 and GSE30122 showed that CASP3 and PTGES were significantly upregulated, whereas SLC6A2 was downregulated in DKD, leading to their designation as biomarkers (P < 0.01) (**Figure 2D**).

ROC analysis demonstrated that CASP3, PTGES, and SLC6A2 exhibited good diagnostic performance in both datasets, with AUC values > 0.7 (**Figure 2E**). Among these, CASP3 was a predicted target of DMP and DOP; PTGES of DOP; and SLC6A2 of DMP and DEP.

### 3.2 Nomogram-based predictive modeling

**Figure 3:**
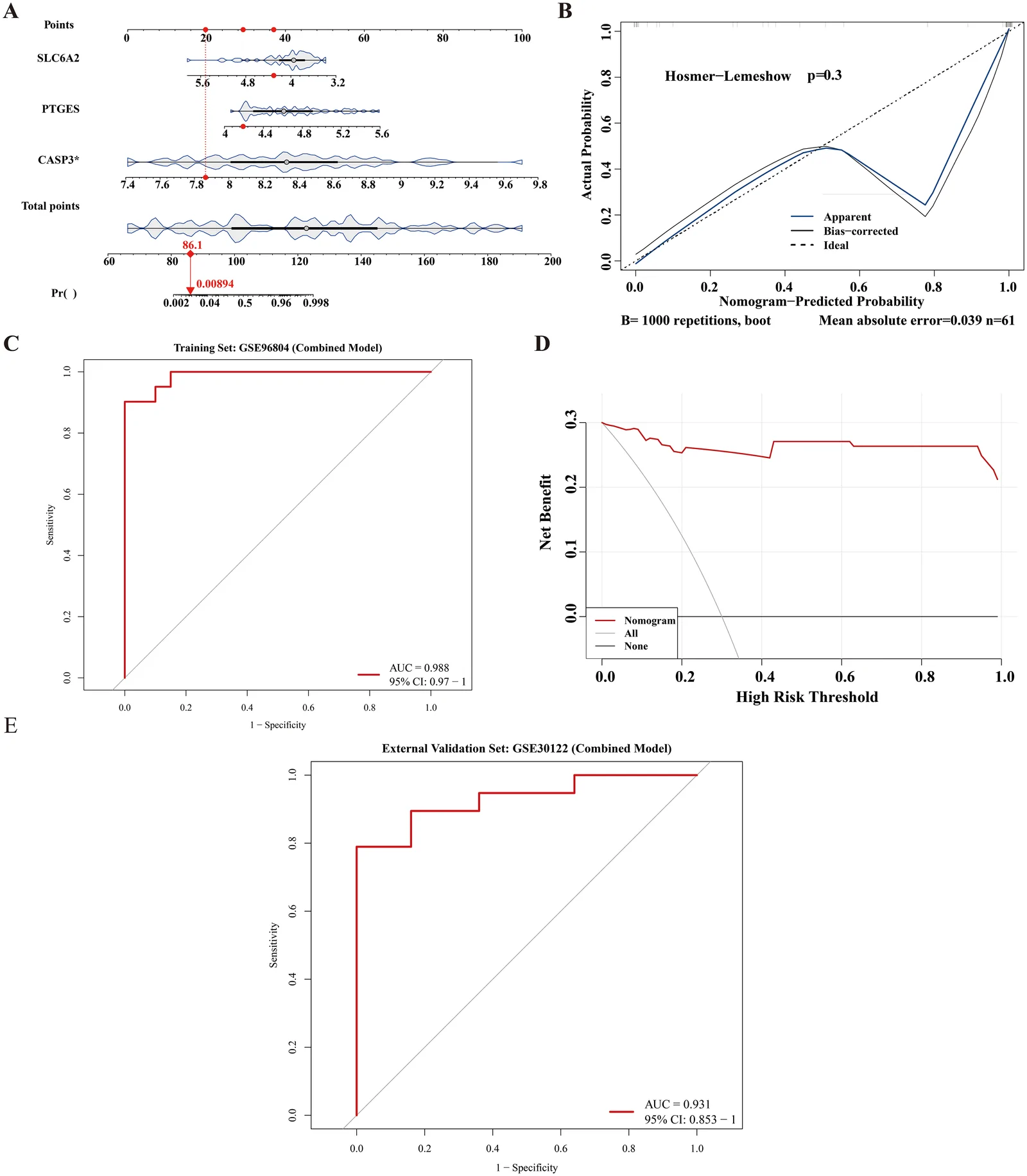
Construction and validation of the biomarker-based nomogram. **(A)** Biomarker nomogram established in the GSE96804 training dataset. The “Points” scale represents the individual score assigned to each biomarker value, while “Total Points” represents the cumulative score obtained from all biomarkers. **(B)** Calibration curve evaluating the agreement between predicted and observed outcomes. **(C)** Receiver operating characteristic (ROC) curve assessing the diagnostic performance of the nomogram in the training cohort. **(D)** Decision curve analysis (DCA) evaluating the clinical utility of the nomogram. **(E)** ROC curve evaluating the predictive performance of the nomogram in the independent validation dataset GSE30122. The horizontal axis represents the false-positive rate (1-specificity), and the vertical axis represents the true-positive rate (sensitivity). ROC, receiver operating characteristic; DCA, decision curve analysis.

In the GSE96804 dataset, a nomogram was constructed based on the identified biomarkers (**Figure 3A**). Calibration analysis showed good agreement with observed outcomes (HL test P = 0.3; **Figure 3B**). The model achieved high discriminative performance (AUC = 0.988, 95% CI: 0.97-1; **Figure 3C**), outperforming individual biomarkers.

Given the limited sample size (41 DKD, 20 controls), internal validation was performed to assess optimism. Bootstrap resampling (1,000 iterations) yielded a mean optimism of 0.022 and an optimism-corrected AUC of 0.966 (**Supplementary Table 7**). Repeated 5-fold cross-validation (50 repeats) further confirmed stable performance (mean AUC = 0.968, SD = 0.061, range: 0.688–1.000). Decision curve analysis indicated improved net benefit over single biomarkers and treat-all/treat-none strategies (**Figure 3D**). External validation in GSE30122 yielded an AUC of 0.931 (95% CI: 0.853-1) (**Figure 3E**), supporting model generalizability.

### 3.3 Functional characteristics of CASP3, PTGES, and SLC6A2

**Figure 4:**
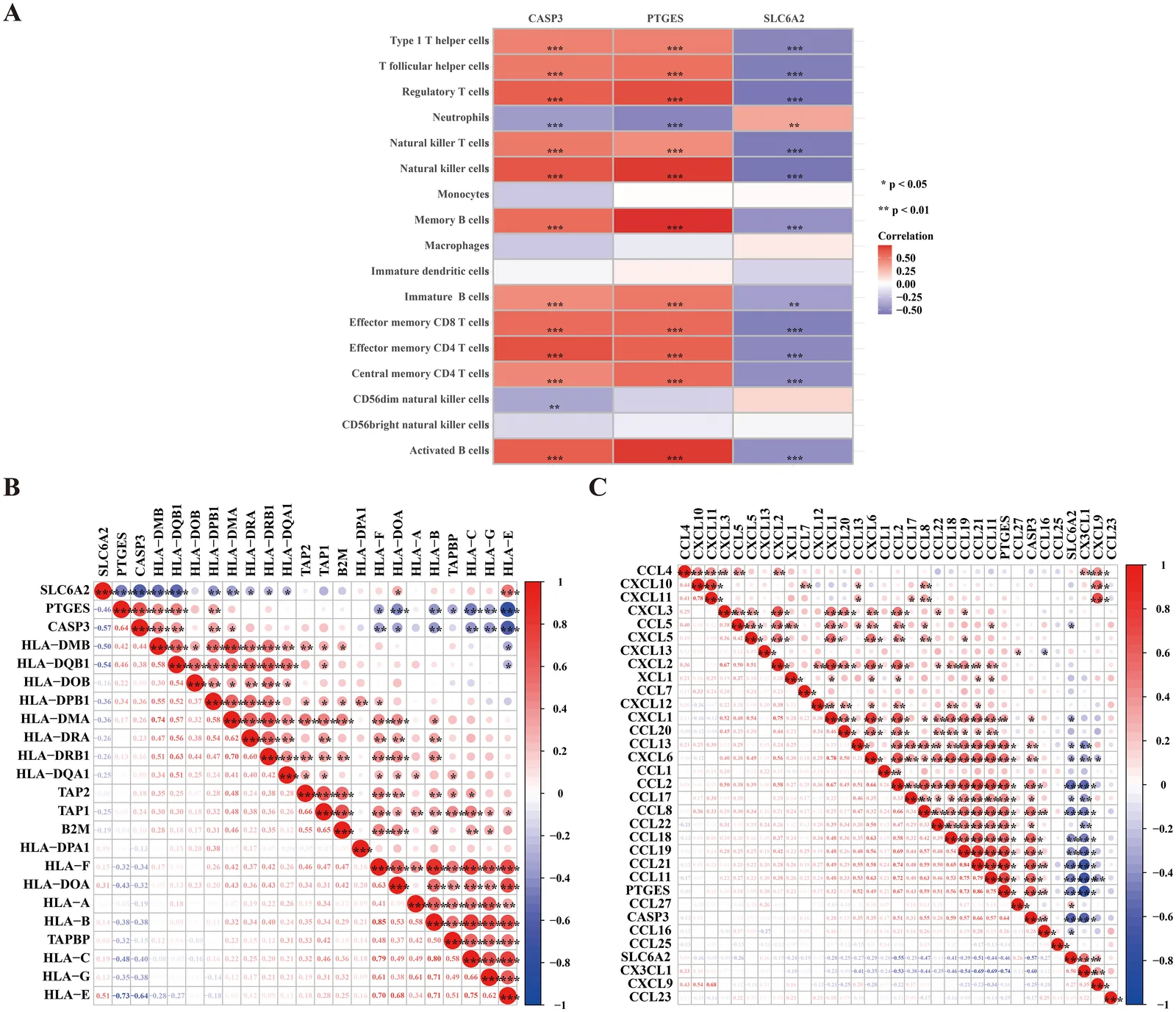
Immune associations of biomarkers. **(A)** Correlation analysis between biomarkers and differentially infiltrating immune cells. **(B)** Correlation analysis between biomarkers and chemokines. **(C)** Correlation analysis between biomarkers and chemokine receptors. The color bar indicates the correlation coefficient, with red representing positive correlations and blue representing negative correlations. Numerical values within the plots represent Spearman correlation coefficients. *P < 0.05, **P < 0.01, ***P < 0.001.

GSEA revealed that genes correlated with CASP3, PTGES, and SLC6A2 were enriched in multiple DKD-related pathways, including olfactory transduction, neuroactive ligand–receptor interaction, ribosome, spliceosome, and oxidative phosphorylation (**Supplementary Figures 2A–C**; **Supplementary Tables 8**–**10**). The three biomarkers displayed largely overlapping pathway patterns, suggesting shared transcriptional responses to the diabetic milieu rather than functional redundancy, as they mediate distinct downstream processes—apoptosis, inflammation, and norepinephrine transport, respectively. Immune infiltration analysis identified significant alterations in 17 immune cell populations in DKD, and the three biomarkers showed distinct associations with immune cell subsets, including effector memory CD4 T cells, memory B cells, and natural killer cells (**Figure 4A**; **Supplementary Figures 3A–C**; **Supplementary Table 11**). PTGES additionally exhibited strong correlations with several immune-related molecules, including CCL21, KDR, ENTPD1, and HLA-E, whereas SLC6A2 was most strongly associated with CXCR1 (**Figures 4B, C**; **Supplementary Figures 3D–F**). These findings represent computationally inferred associations and should be considered hypothesis-generating.

### 3.4 Computational prediction of plasticizer–biomarker binding interactions

**Figure 5:**
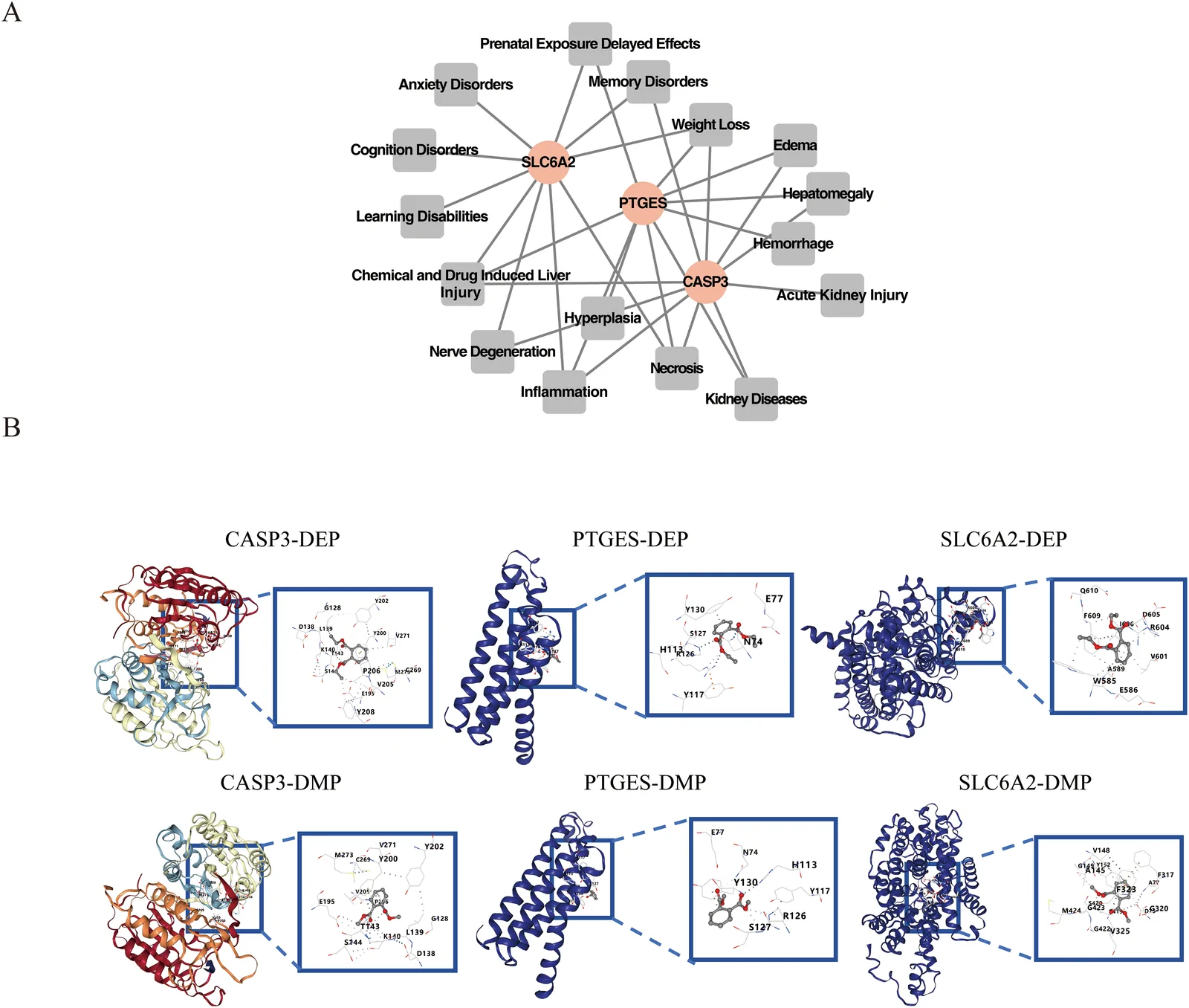
Disease associations and molecular docking analysis. **(A)** Disease association network of CASP3, PTGES, and SLC6A2. **(B)** Representative molecular docking models of CASP3–DEP, PTGES–DMP, and SLC6A2–DEP complexes. The left panel displays the three-dimensional structure of the target proteins, while the right panel shows the corresponding plasticizer molecules. The red-gray ball-and-stick model represents the ligand molecule. Red dashed lines indicate hydrogen bonds, and blue dashed lines indicate hydrophobic interactions. Amino acid residues involved in binding are shown surrounding the ligand, with blue borders highlighting the docking sites. DEP, diethyl phthalate; DMP, dimethyl phthalate.

**Figure 6:**
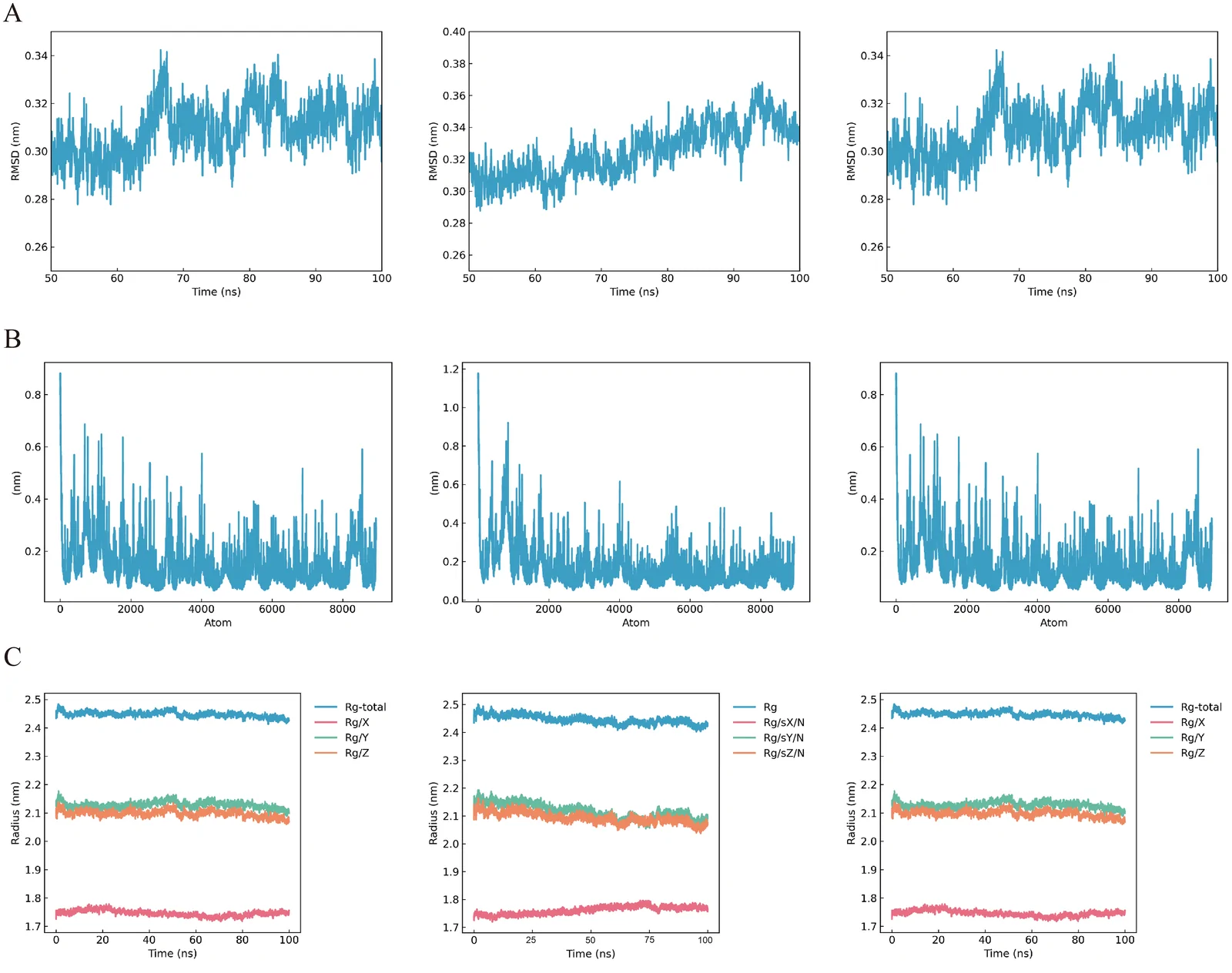
Molecular dynamics simulation of protein–ligand complexes. **(A)** Root mean square deviation (RMSD) trajectories. **(B)** Root mean square fluctuation (RMSF) profiles of amino acid residues. **(C)** Radius of gyration (Rg) analysis reflecting protein structural compactness during the simulation. RMSD, root mean square deviation; RMSF, root mean square fluctuation; Rg, radius of gyration.

Disease association analysis indicated that the identified biomarkers were linked to inflammation, necrosis, weight loss, and chemical- or drug-induced liver injury (**Figure 5A**). Computational docking and molecular dynamics simulations were performed to predict potential interactions; these in silico findings are hypothesis-generating and do not constitute evidence of direct *in vivo* binding. Molecular docking demonstrated favorable binding affinities between DEP/DMP and the three biomarkers, with docking scores ranging from −5.1 to −6.6 kcal/mol (**Figure 5B**; **Table 1**). Among the evaluated complexes, CASP3–DEP (−6.3 kcal/mol), PTGES–DMP (−5.1 kcal/mol), and SLC6A2–DEP (−6.6 kcal/mol) were selected for molecular dynamics simulations. RMSD, RMSF, and radius of gyration analyses indicated stable conformations throughout the 100-ns simulations, with RMSD values remaining within 0.3–0.4 nm (**Figures 6A–C**). Collectively, these computational results suggest that DEP and DMP may form stable interactions with the identified biomarkers.

**Table 1:** Molecular docking results of DMP and DEP with CASP3, PTGES, and SLC6A2.

Gene	Microplastic-associated plasticizers	Vina score	Cavity volume	Center(x, y, z)	Docking size (x, y, z)	Contact residues
CASP3	DEP(Diethyl Phthalate)	-6.3Kcal/mol	2136	3, 3, -24	19, 32, 19	GLY:128:A,ASP:138:A,LEU:139:A,LYS:140:A,THR:143:A,SER:144:A,ARG:167:A,GLU:195:C,TYR:200:C,TYR:202:C,VAL:205:C,PRO:206:C,GLY:207:C,CYS:269:C,VAL:271:C,MET:273:C,GLU:127:B,GLY:128:B,ASP:138:B,LEU:139:B,LYS:140:B,THR:143:B,ARG:167:B,GLU:195:D,TYR:200:D,TYR:202:D,VAL:205:D,PRO:206:D,TYR:208:D,CYS:269:D,VAL:271:D,MET:273:D
PTGES	DEP(Diethyl Phthalate)	-5.1Kcal/mol	138	9, -7, 42	19, 19, 19	ARG:70:A,ARG:73:A,ASN:74:A,GLU:77:A,HIS:113:A,TYR:117:A,ARG:126:A,SER:127:A,TYR:130:A
SLC6A2	DEP(Diethyl Phthalate)	-6.6Kcal/mol	1649	92, 101, 100	30, 19, 19	SER:68:A,GLY:71:A,PHE:72:A,VAL:74:A,ASP:75:A,LEU:76:A,ALA:77:A,ALA:145:A,VAL:148:A,GLY:149:A,TYR:152:A,ASN:153:A,VAL:256:A,PHE:316:A,PHE:317:A,SER:318:A,LEU:319:A,GLY:320:A,PHE:323:A,VAL:325:A,LEU:326:A,ALA:328:A,PHE:329:A,TYR:332:A,ASN:333:A,LEU:415:A,ASP:418:A,SER:419:A,SER:420:A,MET:421:A,GLY:422:A,GLY:423:A,MET:424:A,GLU:425:A,ALA:426:A
CASP3	DMP(Dimethyl Phthalate)	-6.3Kcal/mol	2136	3, 3, -24	18, 32, 18	GLY:128:A,ASP:138:A,LEU:139:A,LYS:140:A,THR:143:A,SER:144:A,ARG:167:A,GLU:195:C,TYR:200:C,TYR:202:C,VAL:205:C,PRO:206:C,CYS:269:C,VAL:271:C,MET:273:C,GLY:128:B,ASP:138:B,LEU:139:B,LYS:140:B,THR:143:B,SER:144:B,ARG:167:B,GLU:195:D,TYR:200:D,TYR:202:D,VAL:205:D,PRO:206:D,TYR:208:D,CYS:269:D,VAL:271:D,MET:273:D
PTGES	DMP(Dimethyl Phthalate)	-5.1Kcal/mol	138	9, -7, 42	18, 18, 18	ARG:70:A,ARG:73:A,ASN:74:A,GLU:77:A,HIS:113:A,TYR:117:A,ARG:126:A,SER:127:A,TYR:130:A,THR:131:A
SLC6A2	DMP(Dimethyl Phthalate)	-6.3Kcal/mol	1649	92, 101, 100	30, 18, 18	GLY:71:A,PHE:72:A,VAL:74:A,ASP:75:A,ALA:77:A,PHE:110:A,ALA:145:A,VAL:148:A,GLY:149:A,TYR:152:A,VAL:263:A,THR:266:A,ALA:267:A,PHE:317:A,SER:318:A,LEU:319:A,GLY:320:A,PHE:323:A,VAL:325:A,LEU:326:A,PHE:329:A,LEU:415:A,ASP:418:A,SER:419:A,SER:420:A,MET:421:A,GLY:422:A,GLY:423:A,MET:424:A

### 3.5 Single-cell transcriptomic characterization of biomarker expression in DKD

**Figure 7:**
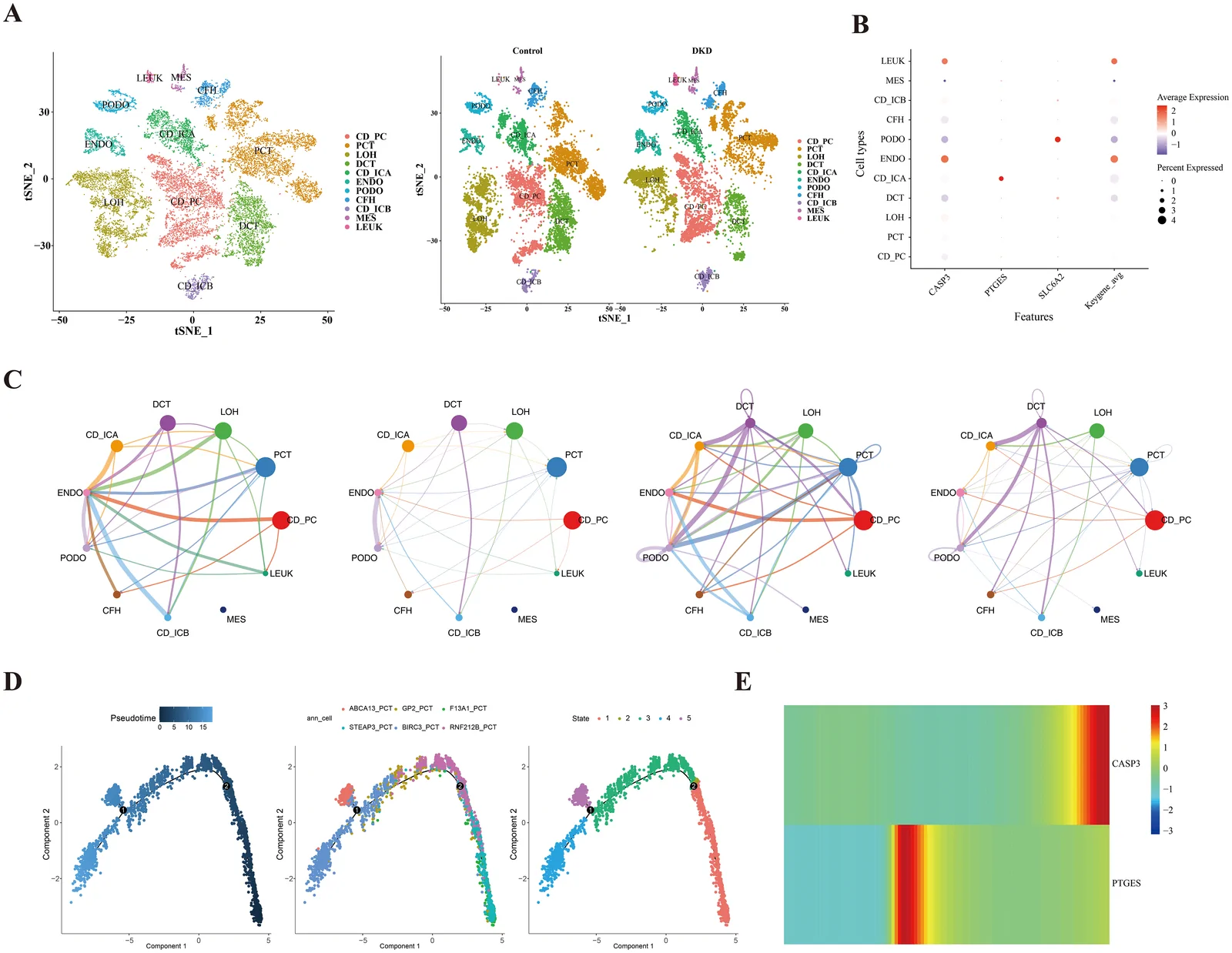
Single-cell transcriptomic analysis of DKD. **(A)** t-SNE visualization of annotated cell populations. **(B)** Expression of marker genes across annotated cell types. **(C)** Cell–cell communication networks in DKD and control groups inferred by CellChat. Node colors indicate cell types, and edge thickness reflects communication strength. (i) the number of interactions in the Control group; (ii) the interaction weights/strength in the Control group; (iii) the number of interactions in the DKD group; (iv) the interaction weights/strength in the DKD group. **(D)**Pseudotime trajectory analysis of PCT cells differentiation states. **(E)** Expression dynamics of CASP3, PTGES, and SLC6A2 along the PCT cells pseudotime trajectory. DKD, diabetic kidney disease; t-SNE, t-distributed stochastic neighbor embedding; PCT, proximal convoluted tubule.

In the GSE131882 single-nucleus RNA-seq dataset (3 DKD and 3 controls), 16,839 high-quality cells and 30,768 genes were retained after quality control. A total of 2,000 highly variable genes were identified, followed by PCA-based dimensionality reduction and clustering, resulting in 22 clusters that were annotated into 11 renal cell types, including proximal convoluted tubule cells (PCT), distal tubule cells, podocytes, endothelial cells, and leukocytes (**Supplementary Figures 4A–D**; **Figure 7A**). Marker gene expression confirmed the accuracy of cell type annotation (**Supplementary Figure 4E**).

Analysis of biomarker expression across cell types revealed that CASP3 was predominantly enriched in PCT cells, which are closely associated with tubular injury and inflammatory responses in DKD (**Figure 7B**). Accordingly, PCT cells were defined as the key cell population for downstream analyses.

Cell–cell communication analysis using CellChat suggested that endothelial cells exhibited the highest overall interaction activity in controls, whereas PCT cells showed increased interaction probabilities in DKD, particularly with podocytes (**Figure 7C**). Ligand–receptor analysis indicated a shift in signaling patterns from FGF-associated interactions in controls to PTHLH–PTH1R–mediated communication in DKD, suggesting disease-associated rewiring of epithelial–glomerular crosstalk (**Supplementary Figure 5A**).

Pseudotime analysis of PCT subclusters showed that CASP3 expression gradually increased toward late pseudotime, PTGES was mainly enriched in intermediate states, and SLC6A2 remained relatively low and stable across trajectories, indicating dynamic but distinct temporal expression patterns of the biomarkers during tubular cell state transitions (**Figures 7D, E**; **Supplementary Figure 5B**). To validate the biological direction of this pseudotime axis, we examined expression dynamics of proximal tubule maturation markers (SLC34A1 and LRP2) and injury markers (HAVCR1 and LCN2) along the trajectory. Maturation markers progressively decreased, whereas injury markers increased along pseudotime. Moreover, DKD cells exhibited significantly higher pseudotime values compared to control cells, supporting their positioning at the injury-prone end of the trajectory (**Supplementary Figures 5C–E**).

### 3.6 DMP and DEP exacerbate tubular injury under high-glucose conditions

**Figure 8:**
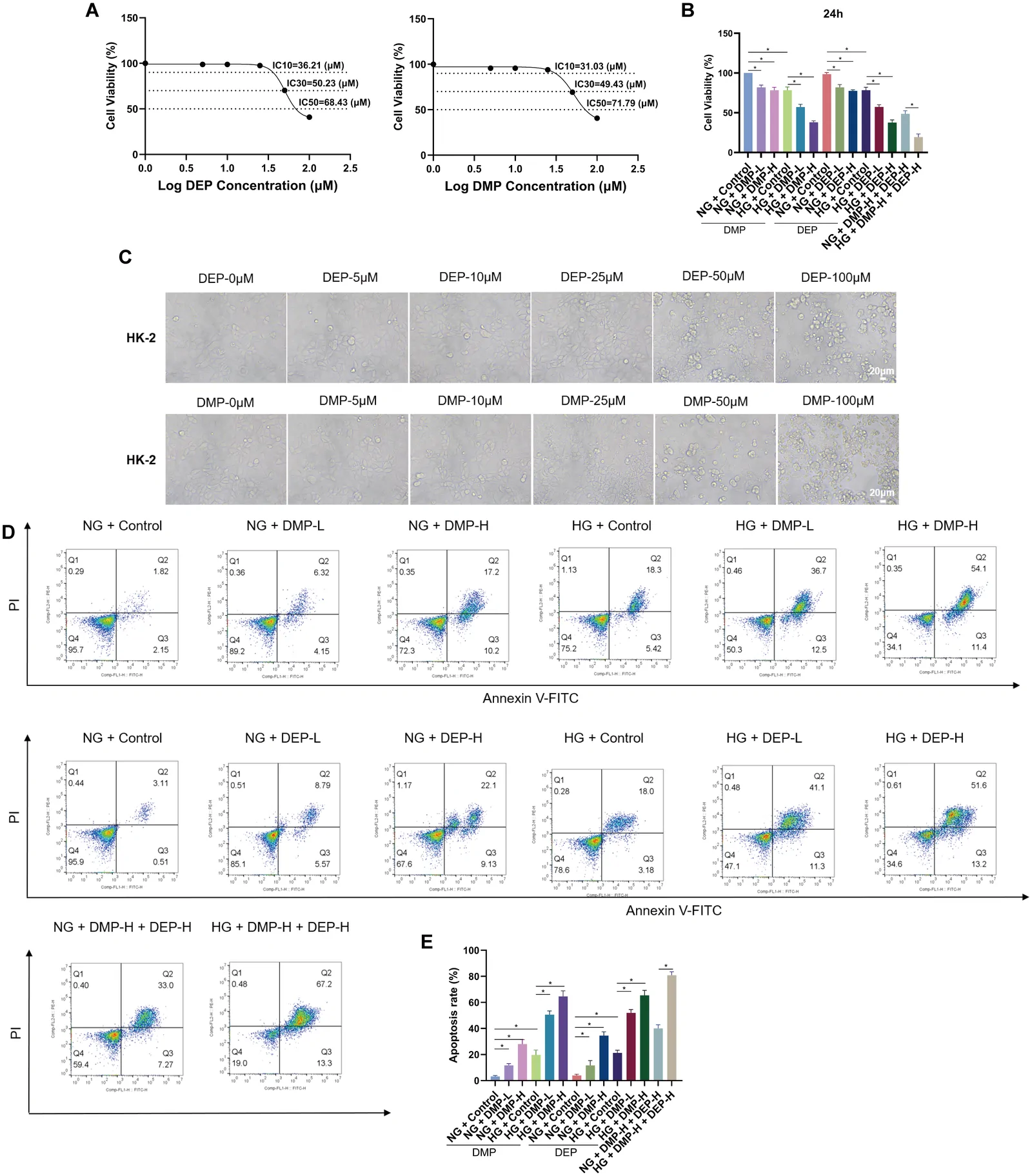
Microplastic-associated plasticizers exacerbate cytotoxicity and apoptosis in HK-2 cells under high-glucose conditions. **(A)** Dose-response curves of HK-2 cells exposed to DMP and DEP. **(B)** Cell viability determined by CCK-8 assay. **(C)** Representative microscopic images of HK-2 cells under different treatments. **(D)** Representative Annexin V-FITC/PI flow cytometry plots. **(E)** Quantification of apoptotic rates. Data are presented as mean ± SD. *P < 0.05, **P < 0.01, ***P < 0.001. NG, normal glucose; HG, high glucose; L, low concentration; H, high concentration. DMP, dimethyl phthalate; DEP, diethyl phthalate; CCK-8, Cell Counting Kit-8; Annexin V-FITC/PI, Annexin V-fluorescein isothiocyanate/propidium iodide; NG, normal glucose; HG, high glucose.

CCK-8 assays revealed that DMP and DEP induced dose-dependent cytotoxicity in HK-2 cells. Based on the calculated IC_50_ values (**Figure 8A**), 10 μM and 30 μM were selected as low (L) and high (H) concentrations for subsequent experiments. These concentrations were selected to elicit detectable acute mechanistic responses within a 24-h exposure paradigm and do not directly model chronic environmental exposure, which occurs at substantially lower levels. While DMP exposure caused a modest reduction in cell viability under normal glucose (NG) conditions, high glucose (HG) treatment significantly amplified this cytotoxicity (**Figures 8B, C**). Notably, DMP aggravated HG-induced injury in a dose-dependent manner. Consistently, combined exposure to DMP-H and DEP-H resulted in a more pronounced viability loss under HG conditions compared to NG conditions.

Flow cytometry further confirmed that this reduced viability was driven by apoptosis (**Figures 8D, E**). Although HG treatment alone elevated apoptotic rates, the addition of DMP significantly exacerbated this effect, with the highest apoptosis observed in the HG + DMP-H group. Similarly, the pro-apoptotic effects of combined DMP-H and DEP-H exposure were markedly enhanced in the HG milieu. Collectively, these findings indicate that a hyperglycemic environment sensitizes HK-2 cells to phthalate-induced apoptotic injury, while the dose-dependent cytotoxic effects of DMP and DEP further support a potential causal relationship between plasticizer exposure and tubular cell injury but require *in vivo* confirmation.

### 3.7 DMP and DEP dysregulate CASP3, PTGES, and SLC6A2 expression in a diabetic-like environment

**Figure 9:**
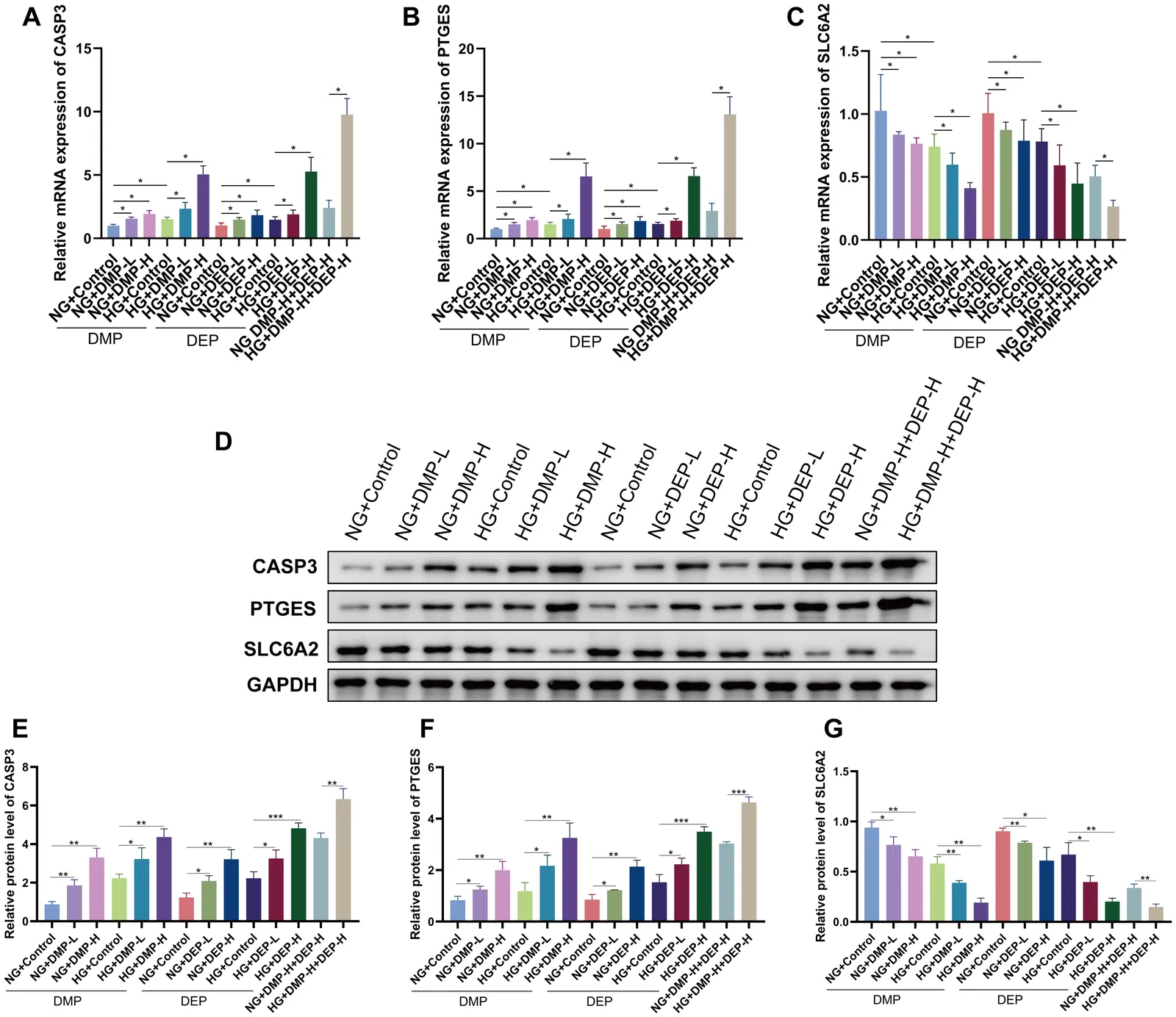
Effects of DMP and DEP on the expression of CASP3, PTGES, and SLC6A2 in HK-2 cells. **(A–C)** Relative mRNA expression levels of CASP3 **(A)**, PTGES **(B)**, and SLC6A2 **(C)** measured by qRT-PCR. **(D)** Representative western blot images showing protein expression levels of CASP3, PTGES, and SLC6A2. **(E–G)** Quantitative analysis of relative protein expression levels of CASP3 **(E)**, PTGES **(F)**, and SLC6A2 **(G)**, normalized to GAPDH. Data are presented as mean ± SD (n = 3). Statistical comparisons were performed using one-way ANOVA. *P < 0.05, **P < 0.01, ***P < 0.001. DMP, dimethyl phthalate; DEP, diethyl phthalate; qRT-PCR, quantitative real-time polymerase chain reaction; GAPDH, glyceraldehyde-3-phosphate dehydrogenase; ANOVA, analysis of variance.

Quantitative RT-PCR verified the expression of the identified biomarkers in HK-2 cells (**Figures 9A–C**). Under NG conditions, DMP exposure upregulated CASP3 (P < 0.05) and PTGES (P < 0.05) while downregulating SLC6A2 (P < 0.05). HG treatment alone induced similar transcriptional shifts; however, the addition of DMP significantly amplified these changes in a dose-dependent manner. Notably, the most pronounced dysregulation occurred following combined exposure to DMP-H and DEP-H under HG conditions, indicating a greater combined effect in the diabetic-like milieu. Western blot analysis corroborated these findings at the protein level, confirming that high glucose exacerbates the phthalate-induced alteration of these key injury markers (**Figures 9D–G**). The concordant expression patterns of CASP3, PTGES, and SLC6A2 between bioinformatic predictions and DMP/DEP-exposed HK-2 cells support the biological relevance of the computational findings in a diabetic-like cellular environment. These *in vitro* results are intended as proof-of-concept evidence under acute high-dose conditions; they do not fully recapitulate chronic, low-dose environmental exposure scenarios.

## 4 Discussion

Plastic pollution has emerged as a major global challenge. Microplastic-associated plasticizers, such as DMP and DEP, are widely distributed in the environment and can enter the human body and exert potential toxic effects independent of the particle carrier (24). A pivotal study confirmed the presence of microplastic-associated plasticizers in both renal tissue and urine, providing the first direct evidence of microplastic-associated plasticizers deposition in human kidneys (25). However, the potential nephrotoxic effects of microplastic-associated plasticizers on renal tissues and cells remain largely unexplored. In this study, target genes associated with both DKD and microplastic-associated plasticizers were identified using publicly available databases. Following machine learning analysis and expression validation, three key biomarkers-CASP3, PTGES, and SLC6A2-were identified. Molecular docking analyses revealed notable binding affinities between DEP and DMP and the identified biomarkers. Furthermore, molecular dynamics simulations confirmed the structural stability of CASP3-DEP, PTGES-DMP, and SLC6A2-DEP complexes. *In vitro* validation confirmed that DMP and DEP exacerbate tubular injury in a diabetic-like environment. Phthalate exposure reduced HK-2 cell viability, increased apoptosis, and dysregulated CASP3, PTGES, and SLC6A2. Notably, these toxic effects were amplified by high glucose, an enhanced combined effect between the diabetic milieu and plasticizer toxicity. Importantly, this study provides associative rather than causal evidence linking microplastic-associated plasticizers to DKD-related renal injury through convergent network toxicology, transcriptomic, and *in vitro* findings.

CASP3 (caspase-3) is a cysteine protease that plays a pivotal role in apoptosis by cleaving specific cellular substrates (26). CASP3-targeted therapeutic strategies have shown promise in mitigating renal injury in DKD patients (27). For instance, administration of the CASP3 inhibitor Z-DEVD-FMK in STZ-induced diabetic mice significantly reduced albuminuria, improved renal function, and attenuated renal fibrosis. These renoprotective effects were linked to the suppression of gasdermin E (GSDME)-dependent secondary necrosis, which suggests a novel mechanism by which CASP3 contributes to DKD progression (28). The *in vitro* results presented here corroborate this functional importance, showing markedly upregulated CASP3 expression in HK-2 cells exposed to DMP/DEP, particularly under high-glucose conditions. Given that CASP3 activation marks irreversible apoptotic execution, the elevated apoptosis and CASP3 expression observed here are more consistent with terminal cell death than reversible cellular stress (29). This suggests that plasticizers may accelerate the apoptotic machinery already primed by the diabetic milieu, consistent with the enhanced apoptotic rates observed in this study. Although CASP3 is not kidney-specific, its consistent upregulation across DKD datasets and experimental models supports its relevance as a marker of apoptosis-driven renal injury.

Prostaglandin E synthase (PTGES) is a critical enzyme which is involved in the biosynthesis of prostaglandin E2 (PGE2) via the arachidonic acid (AA) metabolic pathway (30). Under physiological conditions, PTGES expression is typically low. However, inflammatory stimuli and the presence of cancer cells can rapidly induce its upregulation (31). Studies have shown that PTGES contributes to DKD pathogenesis through multiple mechanisms, including eGFR reduction, tubular cell proliferation, and induction of inflammatory responses (32). In DKD, metabolic disturbances, altered secondary mediators (e.g., angiotensin II and aldosterone), and renal cell injury activate renal inflammation synergistically (33, 34). In the current study, the observed upregulation of PTGES following phthalate exposure supports the notion that these environmental toxicants may fuel the pro-inflammatory state characteristic of DKD. Given the pivotal role of inflammation in DKD pathogenesis, PTGES-mediated renal inflammation may significantly contribute to disease progression. Therefore, its differential expression in DKD is more likely to reflect chronic inflammatory activation than tumor-associated signaling.

SLC6A2 (solute carrier family 6 member 2) is situated on chromosome 16q12.2 and produces the norepinephrine transporter (NET), a key component of neurotransmitter transport systems (35). However, its association with DKD or other renal diseases has not yet been reported. This research provides the earliest evidence linking SLC6A2 to both microplastic exposure and DKD pathogenesis. Patients with CKD commonly exhibit elevated sympathetic nervous activity, characterized by increased norepinephrine levels, heart rate, and blood pressure (36). Chronic sympathetic overactivity induces vasoconstriction and activates the renin-angiotensin-aldosterone system (RAAS), resulting in reduced renal perfusion and tubulointerstitial fibrosis (37). Experimental data revealed a significant downregulation of SLC6A2 under combined high-glucose and phthalate exposure. While its role in renal tubular injury is less characterized compared to apoptosis and inflammation markers, this suppression may reflect a novel disruption in tubular solute transport or sympathetic regulation under combined metabolic and toxicant stress. Although its renal role remains incompletely understood, altered SLC6A2 expression may capture DKD-associated sympathetic dysregulation, a recognized systemic feature of chronic kidney disease. The apparent convergence of GSEA signals across the three biomarkers warrants further comment. While CASP3, PTGES, and SLC6A2 mediate distinct biological processes, their shared enrichment in oxidative phosphorylation pathways likely reflects common upstream cellular stress responses to the diabetic environment rather than functional equivalence. Indeed, mitochondrial dysfunction and impaired oxidative phosphorylation are well-established as fundamental, non-pathway-specific disturbances in DKD (38). This interpretation reconciles the overlapping pathway signatures with the complementary roles of each biomarker and supports their combined use as a multi-marker panel capturing different facets of DKD pathophysiology. Although the discovery dataset was derived from glomerular tissue, validation in proximal tubular cells is mechanistically relevant given the established glomerular–tubular crosstalk that drives DKD progression and the susceptibility of tubular epithelium to xenobiotic injury. Collectively, although CASP3, PTGES, and SLC6A2 are not kidney-specific markers, their reproducible dysregulation across independent datasets, experimental validation, and complementary involvement in apoptosis, inflammation, and neurohumoral regulation support their combined diagnostic value in DKD.

Interestingly, CASP3 and SLC6A2 were associated with neuronal disorders (**Figure 5A**) and showed expression in brain regions including the hippocampus and cerebral cortex (**Figure 7B**). Although seemingly inconsistent with DKD, growing evidence supports kidney-brain molecular similarity. Podocytes share structural, signaling, and transcriptomic features with neurons (39, 40). Thus, the enrichment of CASP3 and SLC6A2 in DKD glomerular datasets may reflect conserved podocyte-neuron biology rather than nonspecific cross-tissue signals, supporting their biological relevance.

Enrichment analysis revealed that pathways associated with oxidative phosphorylation are closely linked to DKD pathogenesis. Under physiological conditions, oxidative phosphorylation serves as the primary source of ATP production in renal tissues (41). Hyperglycemia-induced metabolic stress overloads the oxidative phosphorylation machinery, promoting excessive ROS production and oxidative stress (42). Mitochondrial dysfunction not only impairs ATP generation but also exacerbates ROS accumulation, thereby contributing to renal injury, inflammation, and fibrosis (38). Zhang et al. demonstrated that shifting metabolism from oxidative phosphorylation to glycolysis in tubular epithelial cells elevates renal lactate levels and exacerbates renal fibrosis in DKD (43). The role of oxidative phosphorylation in driving DKD development aligns with the patterns observed in our analysis.

Immune cell recruitment to renal tissue is considered a key mechanism underlying DKD progression (44). The abundance profiles of 17 immune cell subsets differed markedly between DKD and controls, as illustrated by the heatmap analysis. Most differentially abundant immune cells were positively correlated, and the strongest correlation was observed between regulatory T cells and immature B cells. These findings suggest a causal link between circulating immunity and DKD susceptibility, particularly emphasizing the role of B cell subtypes in DKD development (45).

Molecular dynamics simulations provided in silico support for the structural stability of CASP3-DEP, PTGES-DMP, and SLC6A2-DEP complexes. These computational observations suggest that microplastic-associated chemical plasticizers may exacerbate DKD progression by modulating gene expression, promoting inflammation, inducing oxidative stress, and impairing mitochondrial function (11). However, these computational analyses cannot establish biological causality *in vivo*. Beyond the individual biomarkers, the present study highlights a critical interaction between metabolic disease and environmental toxicity. Beyond binding characterization, disrupting plasticizer-biomarker interactions may restore protein function and mitigate DKD injury. Mechanistically, DMP/DEP binding to CASP3 may facilitate caspase-3 activation and downstream PARP-1 cleavage, thereby promoting tubular apoptosis (46). DMP interaction with PTGES may enhance PGE2 biosynthesis through perturbation of the glutathione-dependent catalytic site, amplifying inflammatory signaling (47). Likewise, DEP binding to SLC6A2 may impair NET-mediated norepinephrine transport, contributing to sympathetic dysregulation (48). While requiring enzymatic, functional, and mutagenesis validation, these findings link docking predictions to potential therapeutic actionability. The identified biomarkers may also aid exposure-informed DKD monitoring, whereas PTGES inhibitors, caspase-targeting therapies, and NET modulators warrant investigation as candidate interventions.

The use of a high-glucose tubular epithelial model allowed for the approximation of the cellular stress characteristic of DKD. While hyperglycemia alone induced modest cytotoxicity-consistent with known glucotoxicity-the superimposition of DMP and DEP exposure significantly exacerbated cell injury. In the present *in vitro* model, DMP and DEP were administered in dissolved form, consistent with their predominant post-leaching free-chemical state in real-world environments. This finding supports the hypothesis that the diabetic kidney is sensitized to environmental toxicants. Urinary concentrations of DEP metabolites-monobutyl phthalate (MBP) and monobenzyl phthalate (MBzP)-were previously found to correlate with impaired renal function based on NHANES data (49). These findings provide a mechanistic basis for these epidemiological observations, suggesting that microplastic-associated plasticizers act as a “second hit” to the vulnerable tubular epithelium, amplifying oxidative stress and inflammatory signaling initiated by hyperglycemia. As the concentrations used were intended to elicit detectable acute mechanistic responses within a 24-h exposure paradigm, the findings establish biological plausibility rather than directly reflecting chronic environmental exposure, which requires future low-dose long-term investigation. While, as HK-2 cells model only tubular epithelium, these findings represent proof-of-concept evidence and require validation in more complex DKD systems.

Additionally, the present *in vitro* model employed DMP and DEP in dissolved form, which captures the chemical toxicity of free plasticizers but does not recapitulate the environmentally relevant scenario of co-exposure to microplastic particles and their adsorbed/released additives. In real-world settings, humans are simultaneously exposed to microplastics and associated plasticizers, and accumulating evidence indicates that combined exposure can produce more pronounced toxic effects compared to single exposures, including nephrotoxic, neurotoxic, and enterohepatic effects primarily mediated by oxidative stress and inflammatory pathways (50–52). Thus, the current findings should be interpreted specifically as the chemical toxicity of soluble plasticizers and not extrapolated to the combined toxicity of microplastic particles with adsorbed additives. Investigations of co-exposure toxicity in more physiologically relevant models represent a meaningful direction for future research.

In addition, 11 distinct cell types were annotated in DKD samples in this study. Among these, PCT cells were identified as the key cell population based on prominent CASP3 expression. PCT cells are the predominant proximal tubular epithelial cells and represent a major site of early injury in diabetic kidney disease, where tubular epithelial damage and programmed cell death are key pathological features (53). Elevated CASP3 expression in PCT cells is consistent with caspase-3–mediated apoptotic tubular injury, a well-recognized mechanism in DKD progression (54). Pseudotime analyses have been widely applied to characterize injury-associated transcriptional states in proximal tubular cells in kidney disease. Integration of single-cell, bulk transcriptomic, and *in vitro* evidence is commonly used to reinforce biological interpretation in DKD studies (55, 56). However, the limited donor number warrants cautious interpretation, and validation in larger spatial or single-cell datasets is required.

Several limitations should be acknowledged. First, the single-cell dataset included only three DKD and three control samples and should therefore be considered exploratory. In addition, the relatively permissive percent.mt threshold (<80%), although consistent with the original analytical pipeline, may affect the interpretation of stress- and injury-related markers such as CASP3. Second, the immune infiltration, GSVA, CellChat, and pseudotime results were computationally inferred and require experimental validation. Third, CASP3, PTGES, and SLC6A2 should be regarded as candidate biomarkers rather than causal disease drivers. The transcriptomic datasets lacked individual-level plasticizer exposure data, and the docking and molecular dynamics results represent computational predictions rather than evidence of direct *in vivo* binding. Fourth, experimental validation was limited to immortalized HK-2 cells and soluble DMP/DEP, thereby excluding other renal cell types, tissue-level responses, and the effects of microplastic particles or particle-bound additives. Fifth, the concentrations and 24-h exposure paradigm reflected acute high-dose conditions rather than chronic environmental exposure, and reversibility was not assessed by washout experiments. Moreover, because a mannitol osmotic-control group was not included, the enhanced responses under high-glucose conditions cannot be attributed exclusively to glucose-specific effects. Finally, the lack of *in vivo* validation and co-exposure experiments limits the translational interpretation of these findings. Future studies using chronic low-dose exposure models, organoids, animal models, and exposure-stratified clinical cohorts are warranted.

## 5 Conclusion

This study identified CASP3, PTGES, and SLC6A2 as candidate biomarkers potentially linking exposure to the plasticizers DMP and DEP with DKD progression. Multi-omics analyses and preliminary validation in HK-2 cells showed that DMP and DEP altered the expression of these targets and enhanced cytotoxic responses under high-glucose culture conditions. However, these findings are hypothesis-generating and should be interpreted as biomarker-level associations rather than evidence of causal mediation or direct *in vivo* binding. Moreover, the experimental results reflect the chemical toxicity of DMP and DEP in free, dissolved form and should not be extrapolated to the physical or combined effects of microplastic particles and particle-bound additives. Further validation in chronic exposure models, animal studies, and exposure-stratified human cohorts is required to establish their biological and translational relevance.

## Data Availability

The datasets presented in this study can be found in online repositories. The names of the repository/repositories and accession number(s) can be found in the article/**Supplementary Material**.

## Supplementary material

Supplementary Figure 1 Functional enrichment and toxicity prediction of key genes. **(A)** Top 10 enriched Gene Ontology (GO) terms for the 29 key genes. **(B)** Top 10 enriched Kyoto Encyclopedia of Genes and Genomes (KEGG) pathways associated with the key genes. **(C)** Protein–protein interaction (PPI) network of the key genes. **(D)** ProTox 3.0-predicted organ toxicity profiles of ATBC, DMP, DEP, and DOP. Radar plots indicate the confidence of positive toxicity predictions relative to the average values of comparable compounds. GO, Gene Ontology; KEGG, Kyoto Encyclopedia of Genes and Genomes; PPI, protein–protein interaction; ATBC, acetyl tributyl citrate; DMP, dimethyl phthalate; DEP, diethyl phthalate; DOP, dioctyl phthalate.

Supplementary Figure 2 Gene set enrichment analysis of biomarkers. **(A)** Top 5 enriched pathways for genes positively correlated with CASP3. **(B)** Top 5 enriched pathways for genes positively correlated with PTGES. **(C)** Top 5 enriched pathways for genes positively correlated with SLC6A2. Representative pathways include oxidative phosphorylation, spliceosome, ribosome, and neuroactive ligand–receptor interaction.

Supplementary Figure 3 Extended immune microenvironment analyses. **(A)** Heatmap showing the relative abundance of 28 immune cell populations in DKD and control samples. **(B)** Differential infiltration scores of immune cells between DKD and control groups. **(C)** Correlation matrix of differentially infiltrating immune cells. **(D)** Correlation analysis between biomarkers and immunosuppressive factors. **(E)** Correlation analysis between biomarkers and immunostimulatory factors. **(F)** Correlation analysis between biomarkers and major histocompatibility complex (MHC) molecules. The color bar indicates correlation coefficients, with red representing positive correlations and blue representing negative correlations. DKD, diabetic kidney disease; MHC, major histocompatibility complex.

Supplementary Figure 4 Quality control and cell annotation of single-cell RNA sequencing data. **(A)** Quality control metrics used for cell filtering. **(B)** Identification of highly variable genes (HVGs). **(C)** Principal component analysis (PCA) and selection of significant principal components for downstream analyses. **(D)** Cluster annotation of identified cell populations. **(E)** Expression of marker genes across annotated cell types. The size and color intensity of circles represent the proportion and expression level of marker genes, respectively. HVGs, highly variable genes; PCA, principal component analysis.

Supplementary Figure 5 Extended cell communication and pseudotime analyses. **(A)** Bubble plots depict cell–cell communication inferred from ligand–receptor pairs in control and DKD groups. **(B)** UMAP visualization of plasmacytoid dendritic cell (pDC) subclusters. **(C)** Expression dynamics of proximal tubule maturation markers (SLC34A1, LRP2) and injury markers (HAVCR1, LCN2) along PCT pseudotime trajectory. **(D)** Combined maturation score (mean of SLC34A1 and LRP2) and injury score (mean of HAVCR1 and LCN2). **(E)** Violin plot comparing pseudotime values between control and DKD groups. DKD, diabetic kidney disease; UMAP, uniform manifold approximation and projection; pDC, plasmacytoid dendritic cell; PCT, proximal convoluted tubule.
