# Supplementary material for: Unveiling the molecular toxicity of plasticizers derived from microplastics (DMP and DEP) in diabetic kidney disease: integrative insights from network toxicology and multi-omics analysis
Source: Front Immunol. 2026 Aug 26;17:1918728. doi: 10.3389/fimmu.2026.1918728 (PMC13556295; doi:10.3389/fimmu.2026.1918728)
Supplement: Supplementary file 1 [file SupplementaryFile1.pdf]

frontiers

The International Society for the Advancement of Cytometry (ISAC) has highlighted the importance of including comprehensive methodological information to ensure data reproducibility and reliability. In line with this, Frontiers in Immunology now requires authors to submit a checklist for manuscripts that involve flow or mass cytometry. This checklist helps standardize the reporting process, improving the quality and transparency of published data. By doing so, we support scientific progress, making it easier for other researchers to replicate and validate experiments.

This form should be submitted with any manuscripts using flow or mass cytometry.

Sample/specimen/material description

☐ Total blood

☐ PBMCs

☐ Organ digests

Other HK-2 human proximal tubular epithelial cells

Did the samples suffer any treatment before or after incubation with the antibodies?

☑ Drug Dimethyl phthalate (DMP)、Diethyl phthalate (DEP)

☐ Cell permeabilization ____________________

☐ Dye ____________________

☑ Propidium iodine

☐ Not applicable

Other High-glucose culture condition

Instrument and antibodies

Name of the Cytometer BD FACSCalibur flow cytometer (BD Biosciences, San Jose, CA, USA)

**Antibodies and targets**	**Fluorochrome/ Metal**	**Catalog number/Company**
e.g. anti-CD4	FITC	Cat. XXX/ XXX ltd.
Annexin V	FITC	Cat. CA1020 / Solarbio, China

Propidium iodide	PI	Cat. CA1020/Solarbio,

Data analyses

1. Name of the software FlowJo ______________________________
2. Reference gating strategy in the manuscript or supplementary material

Gating strategy in (eg Figure X) Figure 8D (Annexin V-FITC/PI quadrant gating; early apoptosis: Annexin V /PI ; late apoptosis: Annexin V ______________________________
